# NAT10: a potential factor to reverse tumor chemotherapy resistance and radioresistance (Review)

**DOI:** 10.3389/fimmu.2026.1777527

**Published:** 2026-06-30

**Authors:** Shijiao Li, Yuan Fang, Yixu Fan, Hao Niu, Xia Sun, Zhenyu Xue, Rui Ling, Ye Hua, Xiang Tang

**Affiliations:** 1 Department of Head and Neck & General Oncology, Affiliated Hospital of Jiangsu University, Zhenjiang, Jiangsu, China; 2 Department of Abdominal Oncology, Affiliated Hospital of Jiangsu University, Zhenjiang, Jiangsu, China; 3 Department of Thoracic Oncology, Affiliated Hospital of Jiangsu University, Zhenjiang, Jiangsu, China; 4 Department of Cardiology, Affiliated Hospital of Jiangsu University, Zhenjiang, Jiangsu, China

**Keywords:** cancer drug resistance, chemoresistance, DNA damage repair, NAT10, radioresistance, RNA acetylation

## Abstract

NAT10 (N-acetyltransferase 10) is a nucleolar acetyltransferase with catalytic acetylation function, which is currently the only known acetyltransferase that catalyzes the N4-acetylcytidine (ac4C) modification in RNA. As the “writer” protein for ac4C, NAT10 plays a critical role in various biological and pathological processes. In this article, we first introduce the structural and functional characteristics of NAT10, followed by a comprehensive synthesis of its multifaceted molecular mechanisms orchestrating tumor radiotherapy and chemotherapy resistance. Specifically, we emphasize its roles in hyperactivating DNA damage repair (DDR), driving metabolic reprogramming (including evasion of ferroptosis), and facilitating tumor immune evasion via PD-L1 regulation and T-cell suppression. Furthermore, we critically evaluate the translational implications of NAT10-targeted interventions, highlighting the therapeutic promise of specific inhibitors like Remodelin and the repurposed FDA-approved drug Fludarabine. We believe that understanding the functions and mechanisms of NAT10 in tumors will significantly improve the diagnosis, treatment, and prognosis of cancer.

## 1 Introduction

The conquest of cancer remains profoundly hampered by the development of therapy resistance. While chemotherapy and radiotherapy are cornerstones of cancer treatment, their efficacy is frequently significantly undermined by the emergence of chemoresistance and radioresistance. Acquired drug resistance not only impairs therapeutic efficacy but also constitutes one of the leading causes of treatment failure in various malignancies (1). For instance, historically, systemic chemotherapy has yielded low objective response rates and limited long-term survival benefits in hepatocellular carcinoma (HCC), with the synergistic convergence of multiple drug-resistance mechanisms further severely undermining therapeutic efficacy (2). Similarly, radiotherapy, which exerts its cytotoxic effects primarily by inducing DNA damage, is often circumvented by cancer cells through the aberrant activation of sophisticated DNA damage repair (DDR) pathways (3). This clinical dilemma highlights the need to thoroughly elucidate mechanisms underlying drug resistance.

In recent years, epitranscriptomic regulation—the chemical modification of RNA—has emerged as a pivotal layer of gene expression control that is intimately linked to tumorigenesis and therapeutic response. Among various RNA modifications, ac4C has garnered significant attention for its role in enhancing mRNA stability and translation efficiency (4). NAT10 stands out as the sole known “writer” enzyme responsible for catalyzing ac4C, positioning it as a master regulator at the intersection of RNA metabolism, epigenetic reprogramming, and cellular stress adaptation. Beyond its canonical RNA acetyltransferase function, NAT10 also mediates the post-translational acetylation of protein substrates, further expanding its regulatory repertoire (5). Consequently, NAT10 dysregulation is increasingly implicated in fueling tumorigenesis, metastasis, and, critically, the development of both chemoresistance and radioresistance across diverse cancer types.

Accumulating evidence sketches a complex picture of NAT10’s involvement in therapy resistance. It is reported to exacerbate radioresistance by stabilizing transcripts of DNA repair-related genes via ac4C modification, thereby bolstering the DDR machinery (6). In the context of chemotherapy, NAT10 activity is linked to multidrug resistance, metabolic rewiring, and the suppression of cell death pathways such as ferroptosis (7–9). Promisingly, the pharmacological inhibition of NAT10 (e.g., using compounds like Remodelin or the repurposed drug fludarabine) has shown potential to re-sensitize resistant tumors to conventional therapies in preclinical models, such as by synergizing with cisplatin in bladder cancer organoids (8, 10).

However, the current understanding of NAT10’s role remains fragmented and lacks synthesis. Existing research is scattered across disparate cancer types, often consisting of isolated reports on specific genes or pathways. Several key questions are yet to be adequately addressed: How do the dual functionalities of NAT10 (RNA acetylation vs. protein acetylation) converge to promote resistance? What are the core, conserved pathways within the NAT10 regulatory network (e.g., DDR, metabolic reprogramming, ferroptosis, immune evasion, cancer stemness) that are hijacked in resistant tumors? How does the crosstalk between these pathways underpin the integrated resistant phenotype?

To bridge this critical knowledge gap and provide a unified conceptual framework, we present this comprehensive review. We first delineate the structural characteristics, subcellular dynamics, and core biological functions of NAT10. The central focus of this work is to meticulously dissect and integrate the multifaceted molecular mechanisms through which NAT10 orchestrates tumor resistance. We will systematically examine its involvement in: 1) DNA damage recognition and repair, 2) metabolic reprogramming (glycolysis and lipid metabolism), 3) oxidative stress balance and ferroptosis, 4) cell cycle checkpoint control, and 5) cancer stem cell maintenance. Furthermore, we will evaluate the latest advances in NAT10-targeted inhibitors, discussing their mechanisms of action, preclinical efficacy, and translational potential.

By synthesizing the dispersed evidence into a coherent panorama, this review aims not only to elucidate NAT10’s pivotal role as a central node in therapy resistance but also to underscore its validity as a promising prognostic biomarker and a novel therapeutic target. We envision that the insights consolidated herein will provide a valuable roadmap for future research and inspire innovative combinatorial strategies designed to overcome the enduring challenge of therapy resistance in clinical oncology.

## 2 Overview of NAT10

### 2.1 Structural features

NAT10, a multi-domain protein comprising 1025 amino acids with an approximate molecular weight of 115 kD, belongs to the Gcn5-related N-acetyltransferase (GNAT) superfamily (11, 12). Its functional domains encompass an N-terminal RNA helicase domain (exhibiting ATPase activity), a central GNAT domain (catalyzing acetyltransferase activity), and a C-terminal RNA-binding domain (containing the nucleolar localization signal, NuLS, spanning residues 983–1,025), along with DUF1726 and a predicted tRNA-binding domain (11, 13–15). The C-terminal NuLS domain is essential for nucleolar localization, whereas the GNAT and RNA helicase domains directly participate in RNA modifications (such as ac4C) and interactions with proteins like ACLY (ATP Citrate Lyase) (13, 16, 17). Furthermore, several conserved sites undergo post-translational modifications: K290 (modified by ATAT1-mediated lactylation, influencing RNA acetylation activity), K823 (modified by Khib, regulating protein stability and interaction with USP39), and the C-terminal residues K1016, K1017, and K1020 (modified by PARP1-mediated PARylation, driving nucleolar-to-nucleoplasmic translocation) (13, 18, 19). This underscores the critical dependence of NAT10’s functions on its distinctive multi-domain architecture.

### 2.2 Localization

The subcellular localization of NAT10 is context-dependent. Under physiological conditions, its nucleolar localization is mediated predominantly by the C-terminal NuLS domain, where it participates in the processing of the ribosomal small subunit (SSU) and acetylation of rRNA/tRNA (14, 15, 17, 20). However, in response to DNA damage, chemotherapeutic agents, or during tumor progression, NAT10 can translocate from the nucleolus to the nucleoplasm via PARylation or through interaction with MORC2. This shift enhances its binding to ACLY and modulates the expression of stress-response genes (12, 13, 16). NAT10 localization also varies across different cell types: for instance, in spinal dorsal horn neurons, it is primarily enriched within neuronal nuclei (NeuN-positive cells), while in oocytes, it exhibits dynamic distribution between the nucleolus and nucleoplasm during developmental stages (GV to MII stage) (17, 20). Furthermore, NAT10’s stability is protected by RNPS1 (which inhibits ZSWIM6-mediated ubiquitination and degradation), and its enzymatic activity can be enhanced via allosteric effects induced by long non-coding RNAs (lncRNAs), such as HAAPIR, indicating its functions are subject to multilayered regulation (20, 21).

### 2.3 Function

The core function of NAT10 is to serve as an RNA acetyltransferase “writer” protein, catalyzing ac4C modification to regulate RNA stability and translation efficiency directly. For example, ac4C modification of Vegfa mRNA enhances VEGFA expression in neuropathic pain, thereby promoting cardiac and vascular remodeling (17, 20). Additionally, its acetyltransferase activity extends to proteins (e.g., MORC2), influencing chromatin remodeling and DDR (12). In cancer, elevated NAT10 expression correlates with metastatic progression. Its mechanistic roles involve RNA metabolic reprogramming, ribosome biogenesis, and interactions with RNA-binding proteins such as HNRNPK (15, 19, 22). Notably, although NAT10 contains a tRNA-binding domain, its tRNA/rRNA acetylation requires auxiliary factors (e.g., THUMPD1 and box C/D snoRNAs), indicating the complexity of its functional network (15). Evolutionarily, the structure and function of NAT10 are highly conserved across eukaryotes and archaea, underscoring its central role in fundamental cellular processes (14).

### 2.4 Regulatory mechanisms of NAT10

**Figure 1:**
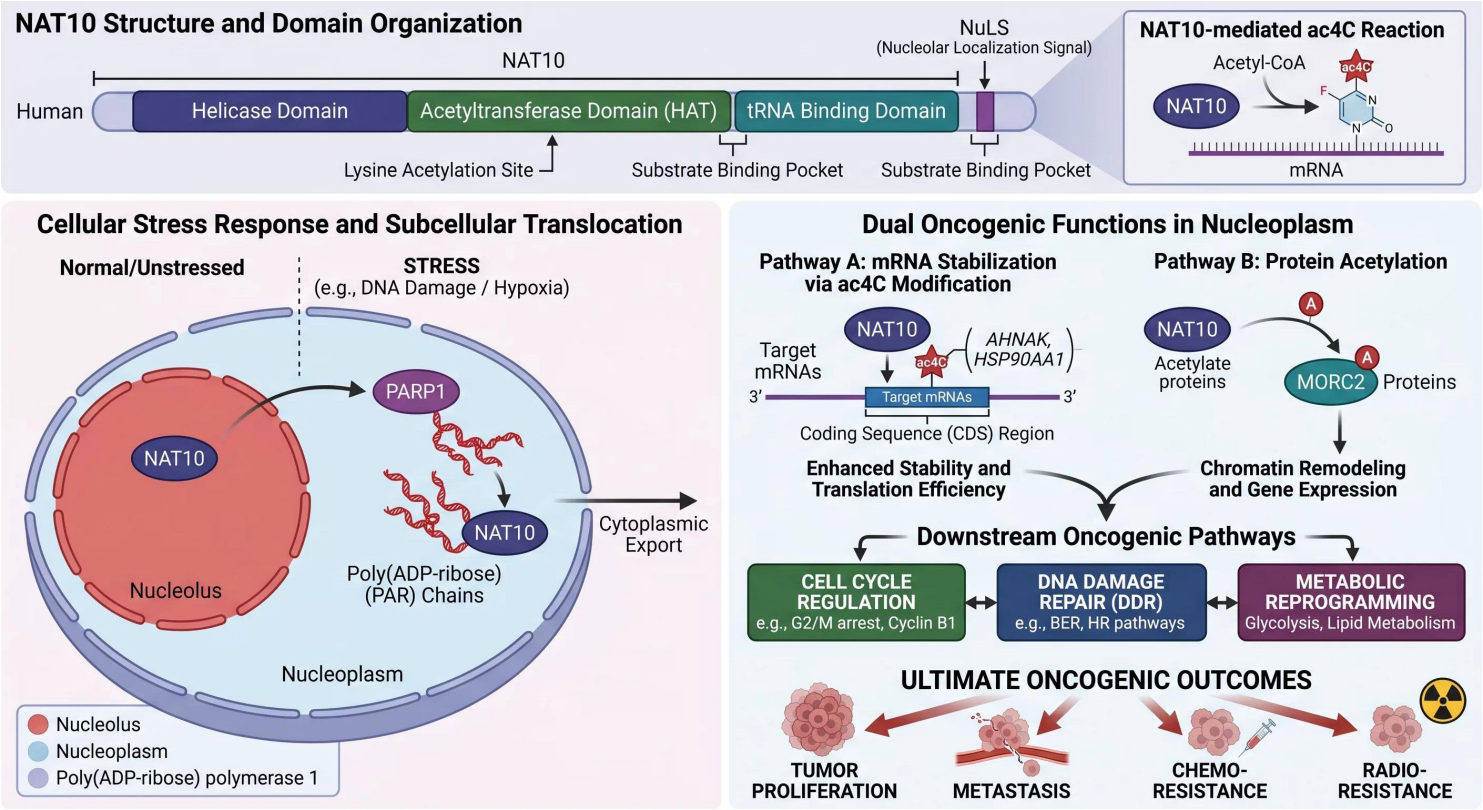
Structural organization, localization, and mechanistic functions of NAT10.

In tumors, NAT10 ac4C modification on RNA, enhancing the stability and translation efficiency of oncogenic target mRNAs, which is enriched in the Coding Sequence (CDS) region, thereby driving malignant tumor progression. NAT10 specifically binds to defined regions of target mRNAs. Its acetyltransferase domain catalyzes cytidine acetylation to promote ac4C modification, which stabilizes mRNA structure and enhances translation efficiency. This process regulates cell cycle progression, apoptosis, metabolism, tumor stemness, immune response, and DDR. Consequently, highly expressed target genes activate downstream signaling pathways, remodel the tumor microenvironment (TME), and ultimately promote tumor proliferation, invasion, metastasis, radioresistance and chemotherapy resistance, and immune evasion (23, 24) (**Figure 1**).

## 3 Common mechanisms of NAT10 in tumor chemoresistance and radioresistance

### 3.1 NAT10 drives resistance via cell cycle regulation and apoptosis evasion

By regulating cell cycle checkpoints and anti-apoptotic signaling networks, NAT10 confers a survival advantage to tumor cells under cytotoxic drug stress.

In HCC and gastric cancer, NAT10-mediated ac4C modification specifically targets the 3’ untranslated region (3’ UTR) of Mdm2 mRNA, significantly enhancing its stability and translation efficiency, which leads to the high expression of the Mdm2 protein (25, 26). This will enable it to acquire oncogenic functions. This subsequently induces significant G2/M phase arrest and drives abnormal proliferation and anti-apoptotic effects. Furthermore, NAT10 can maintain G2/M phase arrest in HCC by upregulating HSP90AA1 to stabilize CDK4 and Cyclin B1; alternatively, it can promote the rapid transition from the G1 phase to the S/G2/M phases by enhancing the translation efficiency of target genes such as CDK1 and CDC6, thereby mediating resistance to lenvatinib (27).

**Figure 2:**
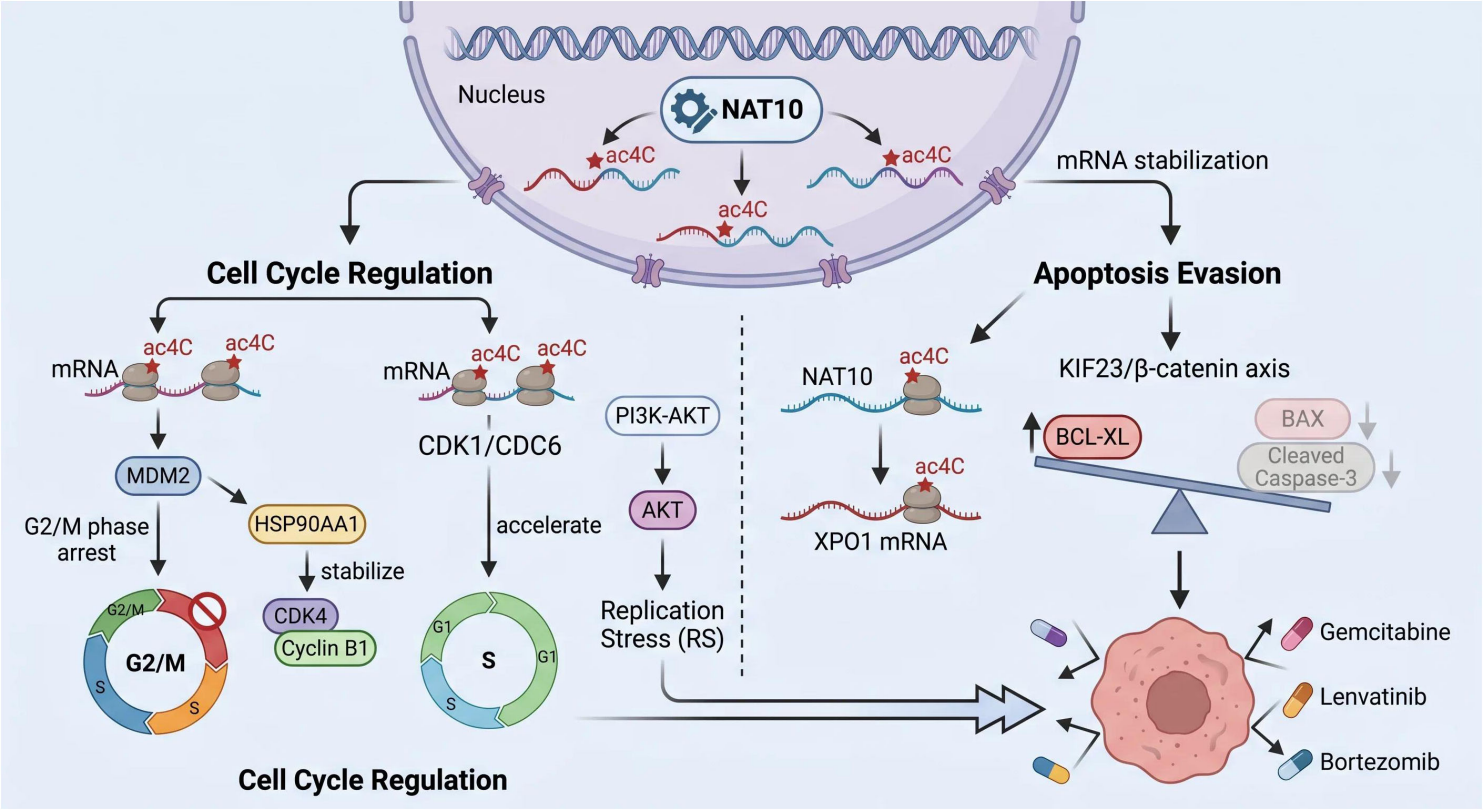
NAT10 drives resistance via cell cycle regulation and apoptosis evasion.

When responding to cell cycle-specific chemotherapeutic agents, NAT10 exhibits multiple mechanisms. In pancreatic cancer (e.g., gemcitabine resistance), high expression of NAT10 is closely associated with the activation of the replication stress (RS) pathway, promoting abnormal cell proliferation via the PI3K-AKT signaling axis (28). Regarding the blockade of apoptosis, NAT10 activates the KIF23/β-catenin axis in colorectal cancer (29); in multiple myeloma, NAT10 can upregulates the classical anti-apoptotic protein BCL-XL and suppresses the expression of the pro-apoptotic factor BAX and cleaved Caspase-3,thereby modulating chemotherapy resistance (30). It can also compromise the cytotoxic efficacy of chemotherapy drugs like bortezomib by enhancing the stability of XPO1 mRNA. Therefore, it is highly likely that NAT10 contributes to chemotherapy resistance by regulating the cell cycle and affecting apoptosis (31) (**Figure 2**).

### 3.2 NAT10 regulates therapy resistance by modulating DNA function

Radiotherapy and chemotherapy primarily eliminate cancer cells by inducing DNA single-strand or double-strand breaks, whereas the aberrant activation of NAT10 significantly enhances the genomic repair capacity of cancer cells. Gene set enrichment analysis (GSEA) confirms that high NAT10 expression is directly associated with the hyperactivation of DDR pathways (32).

At the level of post-translational modifications, radiation- or chemotherapy-induced stress triggers PARP1-mediated poly(ADP-ribosyl)ation (PARylation) of NAT10 at sites K1016/1017/1020, driving the rapid translocation of NAT10 from the nucleolus to the nucleoplasm (13). Once translocated, NAT10 exerts its acetyltransferase activity to acetylate the chromatin remodeling factor MORC2 at the K767 site. This prolongs the G2/M phase and enhances DNA repair capacity by repressing the transcription of CDK1/Cyclin B1, thereby inducing cisplatin resistance and radioresistance in solid tumors such as breast cancer (12). When radiation induces single-strand breaks (SSBs), PARP1 recognizes the DNA damage sites and recruits activation proteins, such as ataxia telangiectasia mutated (ATM) and the phosphorylated histone variant γH2AX. These factors subsequently co-localize with breast cancer type 1 susceptibility protein (BRCA1) at the damage sites to execute DNA repair, thereby establishing radioresistance (13). Concurrently, NAT10 can directly acetylate PARP1 to prolong its half-life and enhance its binding to the XRCC1/LIG3 repair complex. This forms a positive feedback loop that accelerates DNA damage repair and reduces γH2AX accumulation (33). NAT10 deficiency leads to the accumulation of R-loop structures, elevated γH2AX levels, and genomic instability, indicating its intrinsic role in conferring resistance to DNA-damaging agents (34).

At the level of RNA modification, NAT10 targets specific DDR-related transcripts for ac4C modification. For instance, in bladder cancer, it modifies AHNAK mRNA and prevents its degradation by HNRNPQ/THRAP3, thereby activating the “cisplatin-NF-κB-NAT10” resistance feedback loop (35). In skin cancer, NAT10 decreases the mRNA stability of DNA damage-binding protein 2 (DDB2) via ac4C modification, which in turn suppresses the global genomic nucleotide excision repair (GG-NER) process. Conversely, NAT10 knockout accelerates the clearance of UVB-induced DNA damage (36).

Beyond radiotherapy and platinum-based drugs, NAT10 also plays a critical role in the response to nucleolar stress induced by topoisomerase inhibitors (such as doxorubicin). Recent studies indicate that doxorubicin triggers the translocation of NAT10 from the nucleolus to the nucleoplasm, where it spatially co-localizes with dual specificity phosphatase 12 (DUSP12). As a downstream substrate of DUSP12, NAT10-mediated RNA acetylation is finely tuned during this stress response to maintain the DNA repair capacity of cancer cells. Notably, in DUSP12-deficient HCC, pharmacological inhibition of NAT10 triggers a profound “synthetic lethality” effect, thereby significantly restoring the sensitivity of HCC cells to doxorubicin (37).

**Figure 3:**
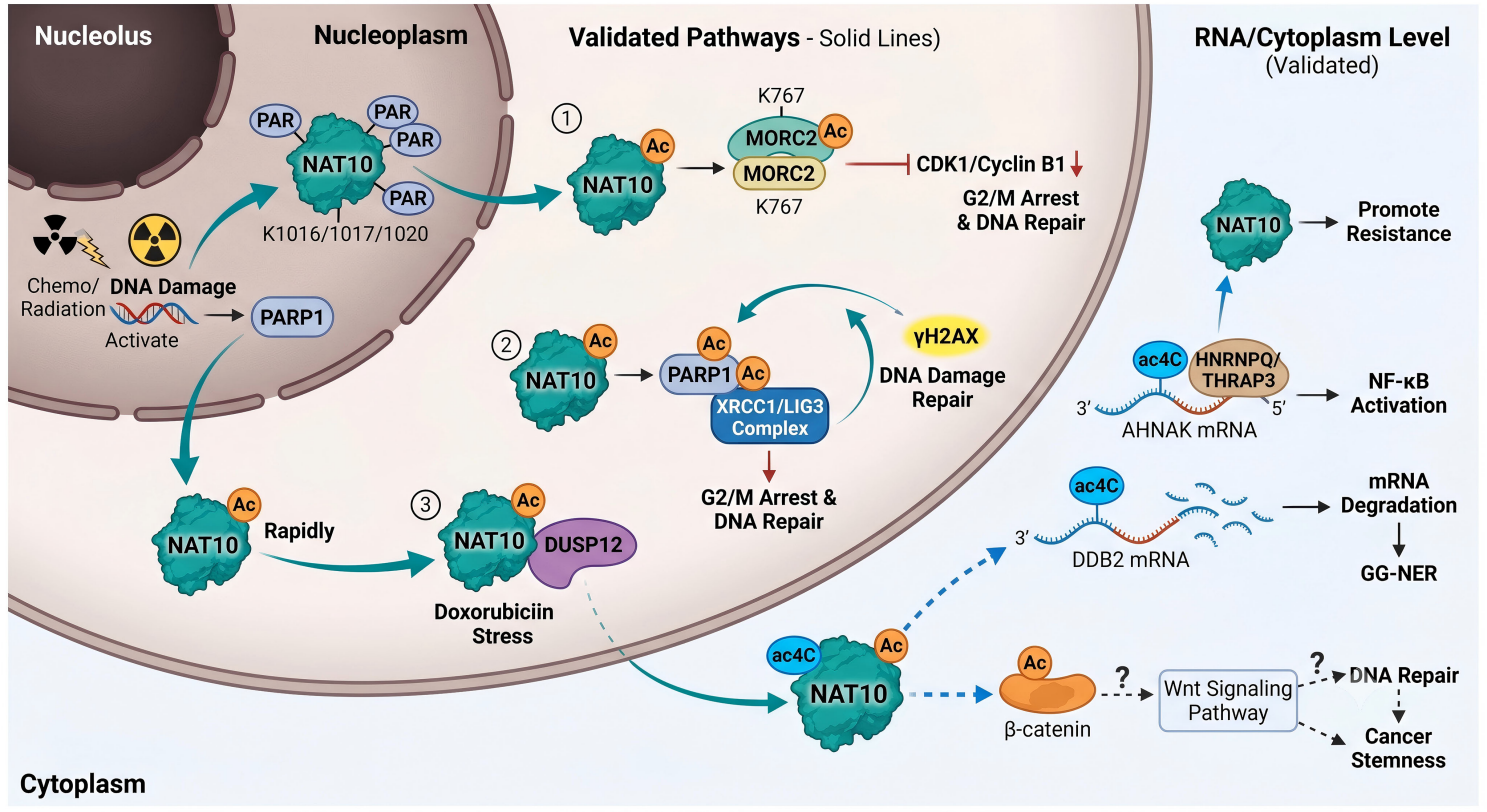
NAT10 regulates therapy resistance by modulating DNA function.

It is important to note that much of the current evidence elucidating NAT10’s mechanistic role in therapy resistance is derived predominantly from *in vitro* cell line models, and extensive *in vivo* validation remains a crucial next step. For instance, NAT10 has been shown to mediate gastric cancer proliferation by acetylating β-catenin to activate the Wnt signaling pathway, which correlates with enhanced cisplatin resistance *in vitro*; however, the precise underlying mechanisms remain to be fully elucidated (38). Given that other studies have established the role of the Wnt/β-catenin pathway in regulating DNA damage repair and stem cell characteristics, we putatively hypothesize that NAT10 may orchestrate these properties via the β-catenin/Wnt axis to mediate chemoresistance (39) (**Figure 3**).

### 3.3 NAT10 drives resistance via metabolic reprogramming

As a core regulator of the epitranscriptome, NAT10 comprehensively reprograms the glycolysis, lipid homeostasis, and amino acid metabolism of cancer cells, providing the essential energetic and material basis for the development of chemo- and radioresistance.

In terms of glycolysis, NAT10 targets and stabilizes JunB mRNA to indirectly upregulate LDHA (observed in breast cancer), or directly mediates ac4C modification of hexokinase 2 (HK2) mRNA (observed in oral squamous cell carcinoma), thereby dramatically exacerbating the “Warburg effect” (40–42). The elevated metabolic flux and massive lactate secretion not only provide a metabolic barrier for cancer cells against radiation-induced oxidative damage, but the resulting acidic TME also enhances chemoradiotherapy resistance and impairs tumor immunity. It achieves this by activating HIF-1α pro-survival signaling, promoting angiogenesis (e.g., VEGF release), inhibiting apoptosis, suppressing the anti-tumor function of T cells (reducing IFN-γ secretion), and recruiting Treg cells (41). Furthermore, studies have shown that by catalyzing the conversion of pyruvate to lactate, LDHA exacerbates TME acidification, enhances DNA repair capacity, and mitigates radiation-induced oxidative damage, thereby conferring radioresistance (43, 44).

Regarding lipid and antioxidant metabolism, NAT10 stabilizes lipid metabolism-related genes such as ACSL3/4, ELOVL6, and ACOT7 via ac4C modification, maintaining the dynamic balance between fatty acid oxidation and synthesis, and effectively resisting lipid peroxidation-induced ferroptosis in ovarian cancer (45–47). In non-small cell lung cancer (NSCLC), NAT10 specifically stabilizes the mRNAs of fatty acid transporters FATP4 and CPT1A, remodeling lipid uptake capacity and thus conferring robust resistance to EGFR-TKIs in cancer cells (NAT10-mediated lipid metabolic reprogramming drives EGFR-TKI resistance in non-small cell lung cancer via ac4C-dependent mRNA stabilization) (48). Additionally, NAT10 upregulates SLC7A11 and GCLC to promote glutathione synthesis, effectively neutralizing the intense oxidative stress induced by chemotherapeutic agents (9, 49).

In metabolism-driven epigenetic remodeling, NAT10, following nucleocytoplasmic translocation, directly acetylates ACLY at K468, resulting in a profound enrichment of nuclear acetyl-CoA. This subsequently triggers the global activation of transcription for key drug-resistance genes, such as CYP2C9 and PIK3R1, via H3K27ac histone modification (16). In acute myeloid leukemia (AML), NAT10 remodels serine metabolism by stabilizing SLC1A4 (MENIN), thereby maintaining the survival advantage of leukemia stem cells (LSCs) (8).

Ferroptosis, a form of programmed cell death driven by iron-dependent lipid peroxidation, plays a pivotal role in determining the radiosensitivity of tumors. Radiotherapy primarily eradicates tumor cells by inducing the massive accumulation of reactive oxygen species (ROS) via ionizing radiation (IR). Conversely, tumor cells acquire radioresistance by activating antioxidant defense systems, such as evading ferroptosis (50). Emerging evidence suggests that NAT10 acts as a core epigenetic regulator within this antioxidant defense network.

In colorectal cancer (CRC), NAT10 exerts potent antioxidant effects by stabilizing the mRNA of ferroptosis suppressor protein 1 (FSP1). As a core ferroptosis inhibitor independent of the glutathione (GSH) pathway, FSP1 effectively scavenges lipid radicals and halts the lipid peroxidation (LPO) cascade. Studies demonstrate that NAT10 knockdown leads to the downregulation of FSP1, which subsequently triggers the characteristic rupture of mitochondrial cristae. This morphological change is accompanied by a profound accumulation of lipid ROS, malondialdehyde (MDA), and labile iron pools, ultimately reversing the radioresistant phenotype of CRC (51, 52).

Similarly, in breast cancer, NAT10 blocks radiotherapy-induced ferroptosis through the canonical pathway. Clinical data reveal a significant positive correlation between the expression of NAT10 and SLC7A11, a key cystine/glutamate antiporter. This mechanism ensures continuous cystine uptake and GSH synthesis, thereby potently suppressing the accumulation of lipid peroxidation products (e.g., MDA and 4-HNE) during radiotherapy. Pharmacological inhibition of NAT10 using specific inhibitors (e.g., Remodelin) effectively depletes GSH and reignites ferroptosis (49).

Recent studies have confirmed that amplifying radiotherapy-induced lipid peroxidation using radiosensitizers (e.g., AGuIX nanoparticles or DNAzyme systems) is an efficacious strategy to overcome radioresistance (53–55). Integrating these mechanisms, NAT10 acts as a crucial upstream shield that allows tumor cells to evade radiotherapy-induced ferroptosis. Therefore, targeted inhibition of NAT10 represents a promising frontier therapeutic strategy to disrupt redox homeostasis and reverse tumor radioresistance.

**Figure 4:**
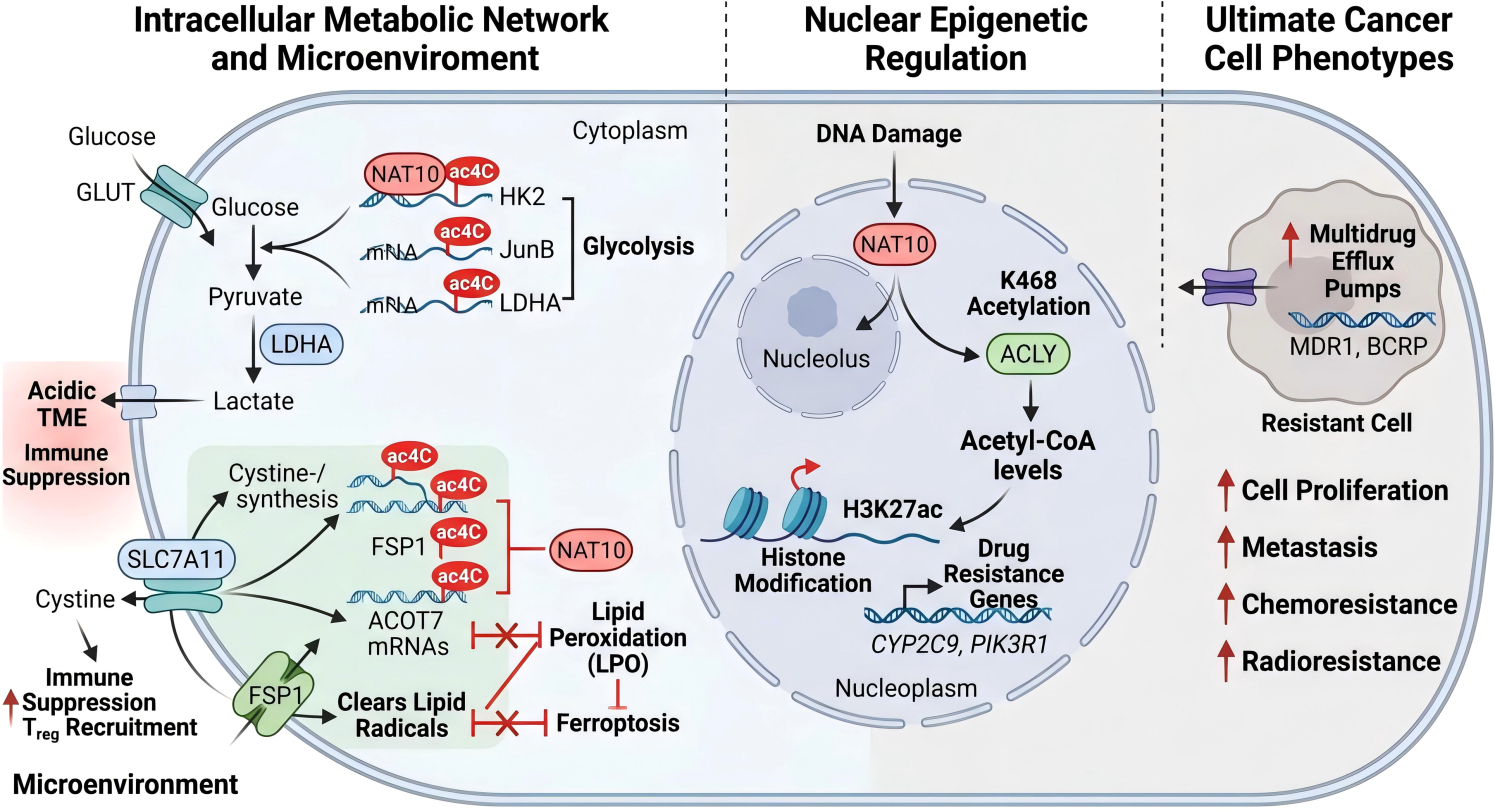
NAT10 drives resistance via metabolic reprogramming.

The radiosensitizer AGuIX nanoparticles reverse radioresistance by inducing the accumulation of lipid peroxidation products (e.g., malondialdehyde [MDA], 4-hydroxynonenal [4-HNE]) (53). Similarly, radioresistant breast cancer cells (e.g., T-47D_RR) maintain redox homeostasis by enhancing their antioxidant capacity, whereas ferroptosis inducers (e.g., SCs) overcome this resistance by catalyzing phospholipid peroxidation (54). Furthermore, a nanotherapeutic system integrating ferroptosis induction and gene regulation—DNAzyme-Fe-HA—attenuates radioresistance by amplifying radiotherapy-induced ferroptosis. This system stabilizes p53 to suppress SLC7A11 expression and block cystine uptake (53). The mechanisms of action of these radiosensitizers align with the aforementioned findings, wherein the inhibition of NAT10 triggers ferroptosis through the significant accumulation of ROS, MDA, and 4-HNE (**Figure 4**).

### 3.4 NAT10 drives tumor resistance via immune microenvironment remodeling and immune evasion

Given the critical role of the tumor microenvironment (TME) in determining therapeutic responses, emerging evidence indicates that NAT10 acts as a key regulator of immune evasion, tightly linking acquired chemo/radioresistance with profound immunosuppression. In cervical cancer, pan-cancer (primarily validated in solid tumors such as lung cancer), and breast cancer models, NAT10 establishes the foundation for immunosuppression chiefly by deteriorating the physicochemical properties of the TME and blocking endogenous immune-activating signals. Specifically, in cervical cancer, NAT10 stabilizes the mRNA of the transcription factor FOXP1 via ac4C modification and promotes its translation, thereby activating hypoxia-adaptive glycolysis (the Warburg effect) in tumor cells; this metabolic shift rapidly consumes glucose and accumulates large amounts of lactate in the microenvironment, depriving peripheral T cells of essential nutrients (56). This metabolic barrier can be reversed through targeted NAT10 degradation using a novel PROTAC degrader (NP1192), which effectively suppresses hypoxic glycolysis and reinvigorates CD8^+^ effector T-cell function (57). In pan-cancer and lung cancer models, the aberrant hyperactivity of endogenous NAT10 activates the MYC/CDK2/DNMT1 axis, leveraging DNA methyltransferase 1 (DNMT1) to forcefully silence the tumor’s intrinsic type I interferon (Type I IFN) response, which cuts off the natural pathway through which tumors release interferons to recruit immune cells, plunging the microenvironment into a thoroughly “cold tumor” state (58). Regarding breast cancer models, on one hand, a cross-talk malignant positive feedback loop exists between NAT10 and histone deacetylase 4 (HDAC4), where the RNA acetyltransferase and protein deacetylase exhibit mutual causality and reciprocal promotion, locking both into sustained high expression in tumor tissues and providing continuous momentum (59); on the other hand, in triple-negative breast cancer (TNBC), NAT10-driven metabolic reprogramming directly impairs immune cell function. Through the ac4C/JunB axis, NAT10 overactivates glycolysis and upregulates lactate dehydrogenase A (LDHA) expression, promoting massive lactate secretion that acidifies the TME. The resulting metabolic acidification forms a formidable immunosuppressive barrier that not only dampens the infiltration and cytotoxic activity of effector T cells (evidenced by reduced IFN-γ secretion) but also facilitates the recruitment of regulatory T cells (Tregs) (41).

In digestive system malignancies (colorectal, pancreatic, and gastric cancers) and squamous cell carcinomas of the upper digestive/aerodigestive tract (nasopharyngeal and esophageal squamous cell carcinomas), the primary contribution of NAT10 manifests as the utilization of more direct “intercellular communication” and checkpoint regulation strategies to evade immune clearance. In colorectal cancer, the classic immunosuppressive metabolite kynurenine within the microenvironment serves as an upstream signal to activate tumor-intrinsic NAT10. Activated NAT10 then directly catalyzes the ac4C acetylation of PD-L1 mRNA to enhance its stability, causing a surge in surface PD-L1 protein expression and precisely suppressing infiltrating CD8^+^ effector T cells (60). Concurrently, NAT10 has been shown to stabilize the mRNA of the secreted protein DKK2, utilizing extracellularly released DKK2 to obstruct effective T-cell infiltration (61). In pancreatic cancer, a highly representative model of immune-insensitive tumors, studies have pioneered a “Dual Modulation” model, demonstrating that targeted intervention against NAT10 not only maintains the intrinsic tumor ETS2-PD-L1 positive feedback loop and KRT8 expression but also directly improves and restores the RNA metabolic status and effector cytotoxicity of CD8^+^ effector T cells themselves (62). In cisplatin-resistant gastric cancer models, NAT10 exhibits the capacity to directly modulate immune checkpoint pathways to mediate T-cell suppression; it catalyzes the ac4C modification of DUSP1 mRNA to activate the JNK/ERK pathway, which subsequently upregulates the high expression of the immune checkpoint molecule PD-L1 via the transcription factor FOSB, thereby enhancing the resistance of tumor cells to cytotoxic drugs while simultaneously shielding them from T-cell-mediated killing (63). This mechanism of manipulating the microenvironment via extracellular secretory factors is mirrored in nasopharyngeal carcinoma (NPC), a typical squamous cell carcinoma model. Research has established that NAT10 regulates DDX5 through ac4C modification, which in turn modulates the expression or release of high mobility group box 1 (HMGB1)—one of the critical signaling molecules (alarmins) in chemoradiotherapy-induced immunogenic cell death (ICD) of tumors—thereby facilitating the recruitment of immunosuppressive cells (64). Meanwhile, in highly aggressive malignancies such as esophageal squamous cell carcinoma (ESCC), this adverse microenvironment may broadly affect other immune compartments, potentially driving tumor-associated macrophages (TAMs) toward M2-type polarization (65).

**Figure 5:**
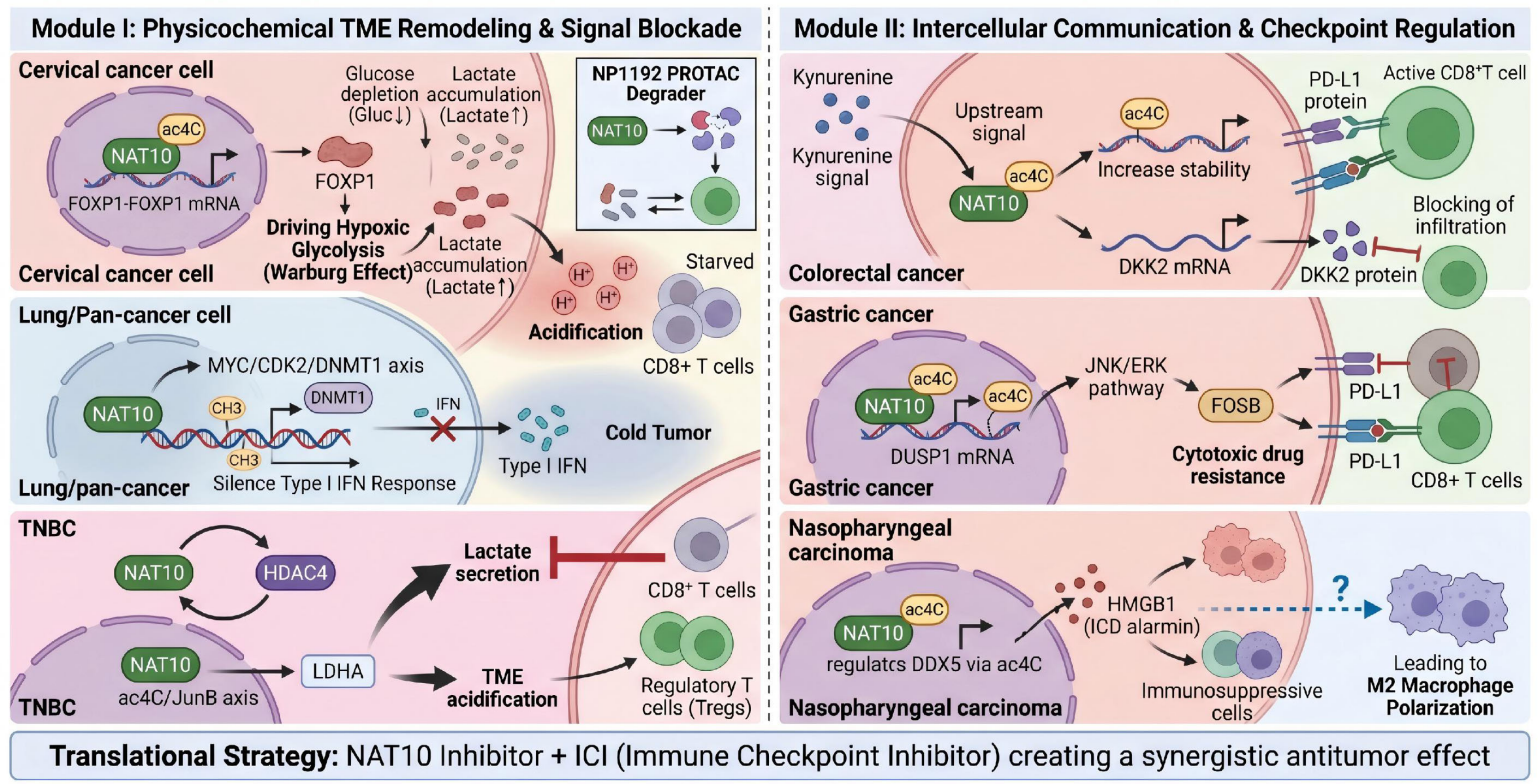
NAT10 drives tumor resistance via immune microenvironment remodeling and immune evasion.

In summary, given the multi-dimensional synergistic roles of NAT10 in intrinsic resistance, chemoradiotherapy resistance, and immune evasion, combining NAT10 inhibitors with immune checkpoint inhibitors (ICIs) represents a highly rational and promising translational therapeutic strategy capable of overcoming multidrug resistance and reinforcing antitumor immunity (59, 66) (**Figure 5**).

### 3.5 NAT10 participate in tumor chemotherapy resistance in other ways

Beyond the aforementioned mechanisms, NAT10 establishes multiple lines of defense for drug resistance across various dimensions, including the maintenance of cancer stem cells, the precise regulation of translational machineries, and the direct promotion of drug efflux.

Cancer stem cells (CSCs) are the root cause of acquired resistance and relapse. By enhancing the stability of relevant stemness target genes, NAT10 directly upregulates stem cell markers and promotes tumorsphere formation. In colon cancer, NAT10 upregulates the expression of stem cell markers and facilitates tumorsphere formation by enhancing target mRNA stability; conversely, knocking down NAT10 significantly suppresses resistance to oxaliplatin and irinotecan (67). In gastric cancer models, NAT10 fortifies the stemness characteristics of CSCs by upregulating the expression of target genes (68). In glioblastoma (GBM), NAT10 enhances the stability of JARID2 mRNA through its mediated ac4C acetylation modification of mRNA, upregulates JARID2 protein expression, and thereby promotes the stemness characteristics and malignant progression of GBM cells (69).At the level of translational regulation, research in esophageal cancer has revealed a pathway through which NAT10 mediates drug resistance: NAT10 not only modifies mRNA but also catalyzes the ac4C modification of a specific subset of tRNAs. Through codon optimality, these modified tRNAs dramatically enhance the translation efficiency of EGFR mRNA, directly mediating the malignant progression of tumor cells and their resistance to gefitinib (70).

**Figure 6:**
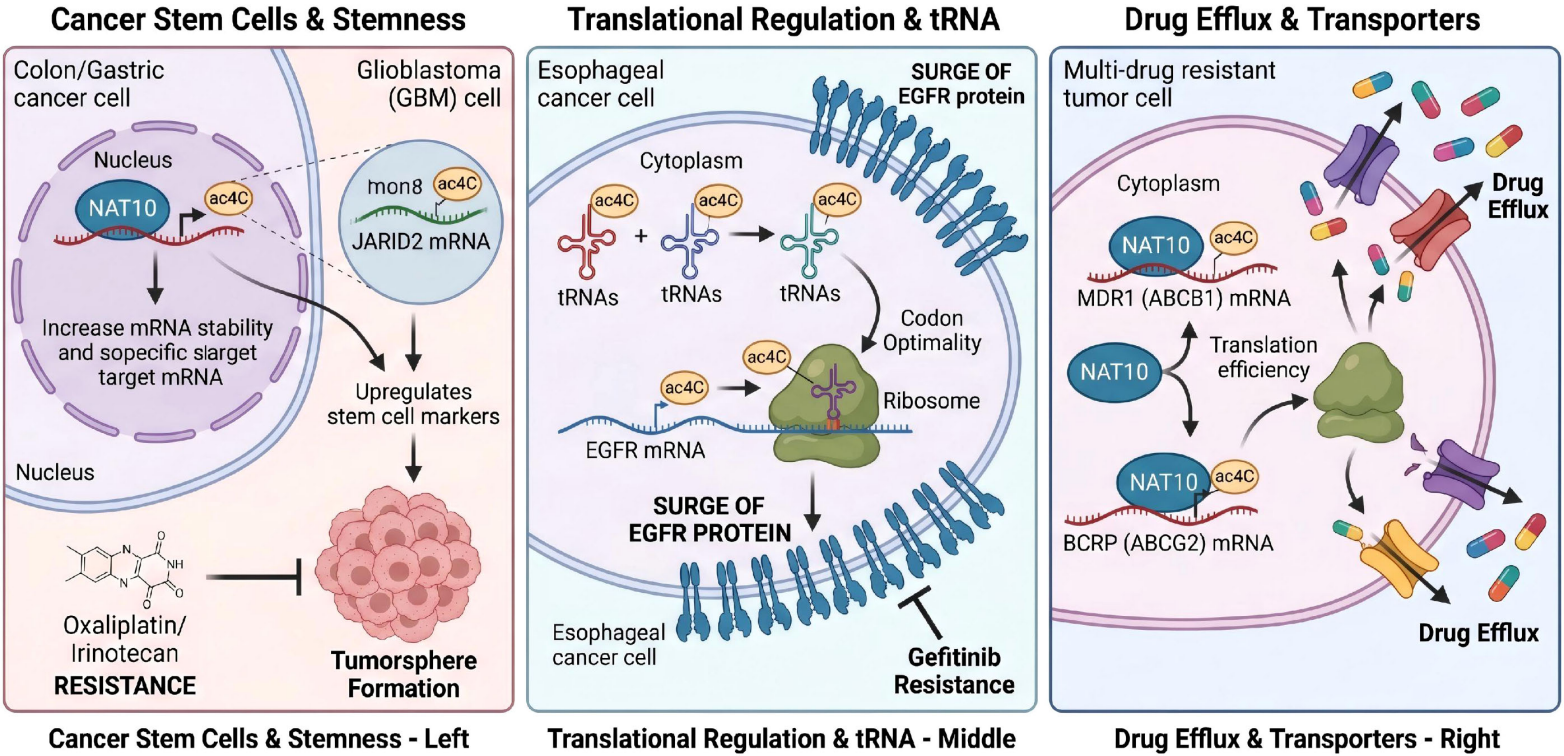
NAT10 participate in tumor chemotherapy resistance in other ways.

Regarding resistance to drug accumulation, NAT10 has been demonstrated to specifically mediate the ac4C modification of mRNAs encoding classical multidrug resistance transporters, namely MDR1 (ABCB1) and BCRP (ABCG2). This modification substantially elevates the translation efficiency of these efflux pumps, which actively extrude chemotherapeutic agents like capecitabine out of the cell, leading to cross-resistance (7)(**Table 1**; **Figure 6**).

**Table 1:** Characteristics, mechanisms, and clinical potential of NAT10 inhibitors.

Inhibitor	Status & original FDA indication	Targeted cancer types	Investigated model systems	Key biological effects	Clinical evidence level & limitations
Remodelin	Synthetic small molecule	HNSCC, Osteosarcoma, Multiple Myeloma, Breast Cancer, Ovarian Cancer, Colorectal Cancer, Nasopharyngeal Carcinoma (NPC)	• In vitro human cancer cell lines (T-47D_RR, MOLM13, etc.)	• Rescues nuclear morphology abnormalities .	Preclinical (In Vitro & In Vivo)
				• Inhibits tumor growth, proliferation, and invasion .	
				• Blocks angiogenesis via inhibiting HIF-1α .	Limitations: Requires therapeutic regimen optimization ; reported potential osteogenic impairment risks ; target specificity remains under academic controversy (debated as a cryptic assay interference chemotype).
				• Reduces ferroptosis .	
				• Suppresses HIV replication.	
Fludarabine	FDA-approved drug	Acute Myeloid Leukemia (AML), Ovarian Cancer	• In silico molecular dynamics (MD) simulations	• Induces G1/S phase arrest and inhibits proliferation .	Preclinical In Vivo Validation & High Translational Potential
	Original Indication: Hematologic malignancies including CLL and NHL		• In vitro human AML cell lines & Patient-derived cells	• Overcomes revumenib resistance in MENIN-mutated AML via serine metabolism reprogramming .	Advantages: FDA approved for repurposing ; excellent structural pocket stability ; high tumor selectivity with low normal cell toxicity.
			• In vivo murine AML xenograft models	• Destabilizes ACOT7 mRNA, suppressing ovarian tumor progression.	
			• In vivo ovarian cancer murine models		
Fosaprepitant	FDA-approved drug	Acute Myeloid Leukemia (AML)	• In silico virtual screening (human NAT10 structural model)	• Significantly suppresses cell growth .	Early Preclinical (In Silico / In Vitro)
	Original Indication: Prevention of Chemotherapy-Induced Nausea and Vomiting (CINV)		• In vitro cell proliferation screening	• Exhibits strong hydrogen-bonding affinity to GLY641 in the acetyl-CoA pocket.	
Leucovorin	FDA-approved drug	Acute Myeloid Leukemia (AML)	• In silico molecular docking	• Marginal effects on cell proliferation .	Weak Preclinical Evidence
	Original Indication: Cytotoxicity mitigation of Methotrexate rescue & Colorectal cancer combination therapy with 5-FU		• In vitro cell screening assays	• Predicted binding affinity to the GLY641 site of NAT10.	Limitations: Limited standalone efficacy for NAT10 inhibition based on current AML model screenings.
Dantrolene	FDA-approved drug	Acute Myeloid Leukemia (AML)	• In silico molecular dynamics (MD) simulations	• Marginal effects on cell proliferation .	Weak Preclinical Evidence
	Original Indication: Management of Malignant Hyperthermia & Spasticity from upper motor neuron disorders		• In vitro cell growth assays	• Identified as a potential pocket binder through human NAT10 screening.	Limitations: Molecular dynamics (MD) simulations indicated higher conformational fluctuations, resulting in lower complex stability.

## 4 Clinical application of NAT10 inhibitors

In laminopathic models, Remodelin was first identified as a specific inhibitor of NAT10. Chemical proteomics using clickable molecular probes coupled with mass spectrometry revealed NAT10 as the sole acetyltransferase target of Remodelin. Circular dichroism (CD) spectroscopy demonstrated that Remodelin’s precursor induces conformational changes in NAT10. Intracellular co-localization assays confirmed probe-NAT10 co-aggregation in the nucleolus, validating direct binding of Remodelin to NAT10. Remodelin is a synthetic small molecule widely recognized as a specific NAT10 inhibitor, featuring a cyclopentenone-thiazol hydrazone chemical core. It competitively binds to the acetyl-CoA binding pocket of NAT10 (key residue G641), thereby inhibiting its acetyltransferase activity toward tubulin. NAT10 serves as a key regulator of nuclear morphology abnormalities. NAT10 inhibition (e.g., by Remodelin or genetic knockout) reorganizes the microtubule network and releases external nuclear membrane forces, consequently rescuing nuclear morphology (71).

Remodelin specifically inhibits the acetyltransferase activity of NAT10 by competitively binding to its acetyl-CoA binding site, resulting in a significant reduction of ac4C modifications on RNA (8, 72, 73). This mechanism demonstrates broad applicability across multiple disease models (8, 74, 75). In tumor models, such as patient-derived xenograft (PDX) models of head and neck squamous cell carcinoma (HNSCC) established in nude mice, oral administration of Remodelin inhibits tumor growth by 55. 7% and downregulates the expression of the oncogene MYC (32). In osteosarcoma and myeloma models, Remodelin suppresses cell proliferation, migration, and invasion by blocking the ac4C-dependent translation of oncogenic proteins such as CEP170 (73, 75). Critically, these studies consistently demonstrate that Remodelin treatment reproduces the effects of NAT10 genetic silencing.

In non-oncological models, Remodelin has been validated as a specific inhibitor of NAT10, too. In cancer and inflammatory diseases, it blocks angiogenesis by inhibiting HIF-1α nuclear translocation; furthermore, genetic depletion of NAT10 via shRNA or CRISPR-Cas9 editing confirmed that Remodelin-mediated HIF suppression requires NAT10 enzymatic activity (76). In renal ischemia-reperfusion injury models, Remodelin reduces ferroptosis markers by suppressing NAT10-mediated ac4C-modulated regulation of NCOA4 mRNA (72). Although genetic validation studies (CRISPR/siRNA) support Remodelin’s target specificity, its therapeutic regimens require further optimization. While HNSCC PDX models demonstrated its safety (32),osteoporosis models indicated potential osteogenic impairment risks (74). Current evidence suggests that its benefits in cancer therapy likely outweigh the risks.

However, scholars contend that remodelin is not a specific inhibitor of NAT10, as direct evidence demonstrating inhibition of NAT10 enzymatic activity remains insufficient. Moreover, the nuclear morphology rescue in Hutchinson-Gilford progeria syndrome (HGPS) models—cited in the seminal study proposing remodelin as a NAT10 inhibitor (71)—was not achieved through NAT10 inhibition but rather stems from remodelin’s assay interference properties. Remodelin has been characterized as a cryptic assay interference chemotype with protein-reactive properties that do not inhibit NAT10’s enzymatic activity. Systematic biochemical, cellular, and biophysical analyses have demonstrated that remodelin does not directly interact with NAT10’s active site and fails to impair NAT10-dependent RNA cytidine acetylation. Furthermore, literature analysis indicates that NAT10 knockdown induces nonspecific cytotoxicity, thereby confounding phenotypic effects attributed to remodelin and creating an artifactual similarity between genetic silencing and pharmacological inhibition (77).

Subsequent *in silico* studies provided counter-evidence by constructing a structural model of human NAT10, revealing through molecular docking and dynamic simulations that Remodelin competitively occupies the acetyl-CoA binding site via hydrogen bonding, hydrophobic interactions, and π-π stacking, thereby inhibiting NAT10’s acetyltransferase activity. Molecular dynamics (MD) simulations confirmed the stability of the Remodelin-NAT10 complex, validating the binding mechanism. Multiple sequence alignment demonstrated 100% conservation of key residues (e.g., GLY639/GLY641) in the acetyl-CoA pocket across 14 species, supporting Remodelin’s cross-species efficacy. Functional assays confirmed that NAT10 inhibition by Remodelin reverses epithelial-mesenchymal transition (EMT) markers (E-cadherin↑/Vimentin↓), reduces HIF-1α expression (counteracting hypoxia), and suppresses HIV replication—consistent with NAT10’s pathological roles. Leveraging the Remodelin-NAT10 complex structure, a virtual screen of 2,115 FDA-approved drugs identified four novel inhibitors: Fosaprepitant, Leucovorin, Fludarabine, and Dantrolene. All four exhibited stronger hydrogen-bonding affinity to GLY641 than Remodelin. Fludarabine demonstrated superior binding indices (e.g., lower binding energy) compared to both Remodelin and the native substrate acetyl-CoA (Ac-CoA), indicating enhanced complex stability. It competitively binds to NAT10’s acetyl-CoA pocket, sharing key residues (VAL631, GLY639, GLY641, LEU719, PHE722) with Remodelin and Ac-CoA. Among the inhibitors, Fludarabine showed optimal structural stability in MD simulations, outperforming Dantrolene (which exhibited higher conformational fluctuations) (78).

While these computational and molecular dynamics models strongly support the feasibility of Remodelin binding to the NAT10 acetyl-CoA pocket, it is imperative to acknowledge that computational evidence cannot entirely substitute for rigorous biochemical and structural biology validation. Therefore, the debate regarding Remodelin’s absolute target specificity versus its potential as a cryptic assay interference chemotype remains an unresolved controversy, avoiding premature settled conclusions until further high-resolution experimental investigations (e.g., Cryo-EM) are conducted.

The description of fludarabine as a NAT10 inhibitor was confirmed. As a purine analogue, fludarabine is intracellularly phosphorylated to its active metabolite F-ara-ATP, which inhibits DNA polymerase and ribonucleotide reductase, thereby blocking DNA synthesis and inducing cancer cell apoptosis. It is currently primarily used for hematologic malignancies such as chronic lymphocytic leukemia (CLL) and non-Hodgkin lymphoma (NHL).

**Figure 7:**
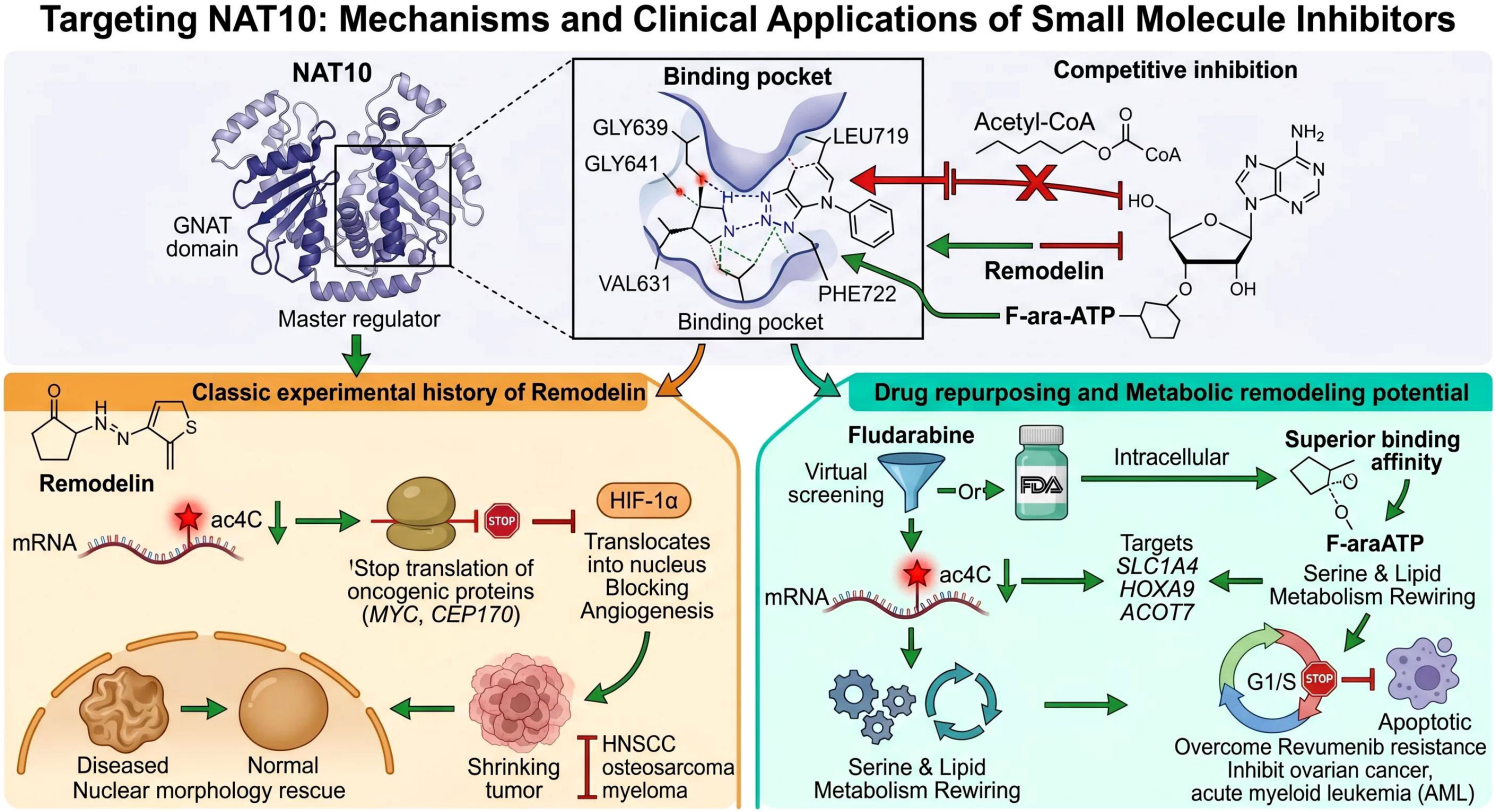
Mechanisms of action and clinical application potential of small molecule NAT10 inhibitors.

Screening of Fosaprepitant, Leucovorin, Fludarabine, and Dantrolene revealed that fludarabine and fosaprepitant significantly suppressed cell growth. Specifically, fludarabine and the established NAT10 inhibitor Remodelin inhibited proliferation across multiple AML cell lines, whereas leucovorin and dantrolene showed only marginal effects. The active metabolite F-ara-ATP significantly reduced cellular RNA ac4C levels, suppressed NAT10 target gene expression (e.g., SLC1A4/HOXA9), decreased total serine levels, and induced G1/S phase arrest in AML cells—effects phenocopying Remodelin. Notably, compared with Remodelin, F-ara-ATP exhibited superior binding affinity to NAT10, as validated by molecular docking and cellular thermal shift assays (CETSA). Both fludarabine and Remodelin suppress AML progression *in vitro* and *in vivo* with high tumor selectivity (low toxicity to normal cells). Critically, they overcome revumenib resistance in MOLM13 cells (a human acute myeloid leukemia cell line) carrying MENIN mutations (e.g., M327I/G331R) by inhibiting NAT10-mediated serine metabolism reprogramming (8). This study first established fludarabine as a NAT10 inhibitor through targeting NAT10-dependent ac4C modification. In ovarian cancer, NAT10 overexpression serves as a diagnostic and prognostic biomarker. Fludarabine inhibited NAT10, destabilized ACOT7 mRNA via suppressing ac4C modification, and blocked the NAT10-ACOT7 axis, thereby suppressing tumor progression (47). As an FDA-approved drug, fludarabine represents a promising repurposed NAT10-targeted therapy for clinical translation (**Table 2**; **Figure 7**).

**Table 2:** Summary of *in vivo* and translational evidence for NAT10 in tumor chemo- and radioresistance.

Cancer type & model systems	Cancer type	Model systems & platforms	Key Molecular targets & mechanisms	Resistant phenotypes & therapeutic outcomes	Caveats & limitations
In Vitro Evidence Note: This tier currently dominates the mechanistic understanding of NAT10-mediated resistance.	Breast Cancer	Cancer cell lines (e.g., T-47D_RR, etc.)	• Mediates post-translational acetylation of MORC2 (at site K767) or PARP1. • Drives ac4C-dependent mRNA stabilization of JunB/LDHA, MDR1/BCRP, and SLC7A11.	• Hyperactivates DNA damage repair (DDR) pathways. • Triggers glycolytic rewiring, drives active chemotherapeutic efflux (capecitabine), and suppresses ferroptosis to evade radiation.	Lacks systemic pharmacokinetic/pharmacodynamic (PK/PD) profiles, complex angiogenesis, and the three-dimensional architecture that governs intratumoral drug penetration.
	Gastric Cancer	• Gastric cancer cell lines	• Stabilizes MDM2 mRNA; targets DUSP1 mRNA via ac4C modification to activate the JNK/ERK pathway. • Acetylates β-catenin to transactivate the Wnt signaling axis.	• Induces G2/M phase arrest and blocks cell apoptosis. • Confers robust cross-resistance to platinum-based drugs (cisplatin).	Regulatory feedback pathways discovered in flat plastic dishes may fail to capture the dense stromal-tumor crosstalk found in vivo.
	CRC	CRC cell lines	• Activates the KIF23/β-catenin axis. • Stabilizes the mRNAs of ferroptosis suppressor protein 1 (FSP1) and secreted protein DKK2 via ac4C acetylation.	• Mediates anti-apoptotic effects and abnormal cell proliferation. • Efficiently scavenges lipid radicals (ROS) to bypass canonical GSH-dependent pathways, granting radioresistance via ferroptosis evasion.	Fails to reflect the systemic influences of gut microbiota, systemic nutrient deprivation, and active immune surveillance on ferroptosis sensitivity.
	Other Malignancies	Multiple myeloma, pancreatic, oral squamous, and esophageal cancer cell lines	• Stabilizes XPO1, BCL-XL, and HK2 mRNAs. • Catalyzes specific tRNA ac4C modifications to enhance EGFR codon translation optimality.	• Promotes multi-drug resistance against various cytotoxic and targeted therapies, including bortezomib, gemcitabine, and gefitinib.	Monolayer cultures cannot replicate the profound tissue-specific barriers to therapy, such as the dense desmoplastic stroma in pancreatic cancer or bone marrow niches.
In Vivo Evidence Note: Current preclinical validation heavily relies on immunodeficient models.	HNSCC	Patient-derived xenografts (PDX) established in nude mice	• Downregulates the expression of the master oncogene MYC. • Disrupts the hypoxia-adaptive NAT10/ac4C-SEPT9-HIF-1α feedback loop.	• Oral administration of the small molecule inhibitor Remodelin successfully suppresses in vivo tumor growth by 55.7%. • Blocks angiogenesis by halting HIF-1α nuclear translocation.	Immune System Insufficiency: Because nude mice lack functional T cells, these models cannot evaluate how NAT10 inhibition affects the systemic antitumor immune response or immune cell infiltration.
	AML	Murine leukemia xenograft models	• Suppresses core target genes including SLC1A4/MENIN and HOXA9. • Overturns leukemia stem cell (LSC) survival by dismantling serine metabolism reprogramming.	• Validates that both the repurposed drug Fludarabine (F-ara-ATP) and Remodelin exert potent, highly tumor-selective therapeutic efficacy in vivo. • Successfully overrides revumenib resistance driven by MENIN mutations (M327I/G331R).	Preclinical studies primarily involve acute or short-term treatment regimens; the potential systemic toxicity of long-term NAT10 inhibition on the hematopoietic microenvironment requires further optimization.
Translational Evidence Note: Platforms bridging bench findings with clinical patient datasets and outcomes.	Bladder Cancer	Patient-derived tumor/platinum-resistant organoids (PDOs)	• Mediates ac4C modification of AHNAK mRNA to protect it from HNRNPQ/THRAP3-mediated degradation. • Sustains the "cisplatin-NF-κB-NAT10" resistance feedback loop.	• Confirms in a 3D biomimetic system that genetic or pharmacological targeting of NAT10 profoundly synergizes with cisplatin, fully restoring chemosensitivity.	Though preserving patient-specific cellular heterogeneity, these ex vivo organoids lack functional neurovascular networks, perfusion, and active systemic metabolic clearance.
	Gastrointestinal & Gynecological Cancers, etc.	Patient cohorts, clinical tissue microarrays, and retrospective survival datasets	• Core clinical positive correlations between NAT10 and FSP1/SLC7A11/ACOT7. • DUSP1/PD-L1 co-expression patterns transactivated through the FOSB cascade.	• Establishes high NAT10 expression as an independent, statistically robust diagnostic and unfavorable prognostic biomarker tied to severely compromised overall survival (OS) and clinical chemoradiotherapy failure.	Clinical cohort analyses are inherently retrospective and establish mathematical correlation rather than causation; full validation requires testing these clinical axes in immunocompetent, syngeneic animal models.

## 5 Discussion

With the continuous evolution of cancer therapies, chemoradiotherapy resistance remains the primary bottleneck leading to clinical treatment failure. As the only known eukaryotic ac4C “writer” protein to date, NAT10 plays a central role in remodeling the survival advantages of tumor cells and their microenvironment through multidimensional epigenetic and epitranscriptomic regulation. This review systematically summarizes the multiple molecular mechanisms of NAT10 in tumor drug resistance and radioresistance. In particular, it highlights how its dual modification of RNA and proteins profoundly impacts key survival pathways, including the DDR, cell cycle regulation, metabolic reprogramming, and ferroptosis evasion.

Given the central role of the immune system in tumor control, NAT10-mediated treatment resistance is not limited to intrinsic pathways within tumor cells but extends more broadly to the remodeling of the TME. Studies have shown that NAT10-driven glycolytic hyperactivation can lead to microenvironmental acidification, thereby inhibiting IFN-γ secretion by effector T cells and recruiting regulatory T cells to construct a robust immunosuppressive barrier (41). More direct evidence indicates that in drug-resistant models, NAT10 can enhance the stability of DUSP1 mRNA via ac4C modification, which in turn transactivates the high expression of the immune checkpoint molecule PD-L1 through the JNK/ERK/FOSB cascade (63). This indicates that NAT10 confers resistance to cytotoxic drugs in cancer cells while synergistically promoting tumor immune evasion. In the treatment of highly aggressive solid tumors such as esophageal squamous cell carcinoma (ESCC), NAT10-mediated resistance to targeted therapy and chemoradiotherapy is frequently observed (70). This suggests that targeting NAT10 may be a critical key to reversing multidrug resistance and relieving the immunosuppressive state.

The function of NAT10 exhibits a high degree of dependence on the cellular context and stress conditions. In response to DNA breaks induced by genotoxic drugs or ionizing radiation, NAT10 rapidly undergoes nucleolar-nucleoplasmic translocation, accelerating the DDR process by acetylating PARP1 or MORC2 (12). Regarding cell cycle regulation, NAT10 demonstrates a delicate “double-edged sword” regulatory pattern: it can induce G2/M phase arrest to gain time for DNA repair, while also accelerating cell cycle progression by upregulating specific targets to counteract the selective pressure of targeted therapies. The duality and tissue specificity of these mechanisms emphasize that future strategies targeting NAT10 must incorporate the tumor’s specific genomic mutation background and current immune status.

At the level of clinical translation, although the early-discovered inhibitor compound Remodelin exhibited considerable anti-tumor effects, significant academic controversy remains regarding whether it directly inhibits NAT10 enzymatic activity and whether there are “false-positive” off-target effects caused by assay interference (77). In contrast, Fludarabine, an FDA-approved drug recently identified through high-throughput screening, possesses exceptional targeting and binding affinity for NAT10. Fludarabine not only outperforms Remodelin in binding energy but also significantly reprograms amino acid and lipid metabolic pathways (8). This “drug repurposing” strategy greatly abbreviates the safety validation and clinical development cycle, providing a highly viable candidate for reversing chemoradiotherapy resistance and initiating combinatorial clinical trials.

Although substantial progress has been made in elucidating the mechanisms of NAT10, this review highlights critical gaps in the field that urgently need to be bridged. First, most current studies on NAT10-promoted drug resistance still rely on *in vitro* cell lines or immunodeficient animal models. There is a severe paucity of *in vivo* data comprehensively evaluating the TME remodeling effects of NAT10 inhibitors in immune-competent syngeneic mouse models. Given the profound link between NAT10, upregulated PD-L1 expression, and T cell suppression, there is an urgent need for future *in vivo* experiments to explore the synergistic mechanisms of combining NAT10 inhibitors (e.g., Fludarabine) with immune checkpoint inhibitors (ICIs). Second, within the ferroptosis pathway, NAT10 establishes an antioxidant barrier by stabilizing core regulatory factors (49). Future research should further explore the combined application of NAT10 inhibitors with novel radiosensitizers (such as AGuIX nanomaterials) to definitively break tumor radioresistance by inducing severe lipid peroxidation.

In summary, NAT10 acts as a central hub linking epitranscriptomic modification, cellular metabolic reprogramming, and microenvironmental immunosuppression, making it a crucial molecular target driving tumor chemoradiotherapy resistance. Deeply unraveling its immunoregulatory network and accelerating the clinical translation of highly specific, low-toxicity targeted drugs will pioneer novel strategic avenues to overcome tumor treatment resistance, reverse the immunosuppressive microenvironment, and ultimately improve the prognosis of cancer patients.
