# Correction: NAT10: a potential factor to reverse tumor chemotherapy resistance and radioresistance (Review)

**DOI:** 10.3389/fimmu.2026.1928728

**Published:** 2026-07-15

**Authors:** Frontiers Production Office

**Keywords:** cancer drug resistance, chemoresistance, DNA damage repair, NAT10, radioresistance, RNA acetylation

Affiliation(s) 1Department of Head and Neck & General Oncology, Affiliated Hospital of Jiangsu University, Zhenjiang, Jiangsu, China

2Department of Abdominal Oncology, Affiliated Hospital of Jiangsu University, Zhenjiang, Jiangsu, China

3Department of Thoracic Oncology, Affiliated Hospital of Jiangsu University, Zhenjiang, Jiangsu, China

4Department of Cardiology, Affiliated Hospital of Jiangsu University, Zhenjiang, Jiangsu, China were erroneously omitted from the paper. These affiliation(s) have now been added for author(s) “Li S, Fang Y, Fan Y, Niu H, Sun X, Xue Z, Ling R, Hua Y and Tang X”.

The original version of this article has been updated.

